# ST-HONet: Spatio-Temporal Hierarchical Network for long-horizon bimanual visuomotor imitation

**DOI:** 10.3389/fnbot.2026.1860170

**Published:** 2026-06-12

**Authors:** Xukun Liu, Fengjuan Xie, Kai Xu, Zhenyu Liu, Shenggang Wei, Guangning Li, Xu Sun

**Affiliations:** Northwest Institute of Mechanical and Electrical Engineering, Xianyang, Shaanxi, China

**Keywords:** bimanual manipulation, long-horizon visuomotor imitation learning, robot policy learning, spatio-temporally hierarchical optimization, variational action transformer

## Abstract

**Introduction:**

Learning robust and temporally consistent manipulation policies from long-horizon visual observations remains a fundamental challenge in imitation learning. While recent Transformer-based approaches reduce compounding errors via temporally extended action chunks, most methods rely on deterministic or unimodal action representations, limiting their ability to capture the inherent multimodality of expert demonstrations.

**Methods:**

In this work, we propose ST-HONet, a Spatio-Temporal Hierarchical Network that integrates three adaptive optimization mechanisms operating jointly across spatial and temporal dimensions. ST-HONet formulates policy learning as conditional generation of extended action segments using a Transformer-based conditional variational autoencoder to explicitly model multimodal expert behaviors through a structured latent representation. To ensure stable optimization over long temporal horizons, we introduce a unified spatio-temporal training framework combining adaptive data augmentation, progressive latent regularization, and multi-stage optimization strategies.

**Results:**

We evaluate ST-HONet on RoboTwin 2.0, a large-scale benchmark for long-horizon bimanual manipulation under domain randomization with automatically generated expert demonstrations. Across representative manipulation tasks, ST-HONet achieves consistently higher task success rates compared to baseline models, while incurring only minimal additional computational overhead.

**Discussion:**

These results demonstrate that explicitly modeling multimodality via a structured latent space, combined with joint spatio-temporal training mechanisms, significantly improves policy robustness and temporal consistency in long-horizon visuomotor imitation learning. The minimal computational overhead further supports the practical deployability of ST-HONet in real-world systems.

## 1 Introduction

Robotic manipulation from long-horizon sensory inputs has made significant progress, yet learning policies with temporal coherence and broad generalization remains challenging for long-horizon tasks. Imitation learning (IL) offers a compelling paradigm by enabling agents to leverage expert demonstrations without explicit reward design. However, classical IL methods suffer from error accumulation and distribution shift during long rollouts, limiting their applicability to complex multi-step behaviors.

Recent work has reframed robot control as sequence modeling, with Transformer-based architectures showing particular effectiveness. Predicting temporally extended action chunks rather than individual actions substantially reduces compounding errors and improves stability in long-horizon manipulation. Despite these advances, most existing methods adopt deterministic or unimodal action representations, which fundamentally limits their ability to capture the inherent multimodality of human demonstrations—where identical visual observations may correspond to multiple valid action sequences.

Probabilistic generative models, particularly conditional variational autoencoders (CVAEs), provide a natural mechanism for modeling multimodal action distributions. When combined with sequence models, latent-variable formulations enable expressive long-horizon policies. However, integrating CVAEs with Transformer architectures introduces significant optimization challenges: posterior collapse, unstable training dynamics, and sensitivity to hyperparameters—issues that become pronounced with long-horizon visual inputs and extended action horizons.

In this work, we propose ST-HONet, a spatio-temporally hierarchically optimized network for long-horizon robot imitation learning. The approach formulates policy learning as the generation of temporally extended action segments conditioned on long-horizon observations, combining chunk-based sequence modeling with latent-variable policy representations. At its core, ST-HONet adopts a Transformer-based conditional variational autoencoder (CVAE) that captures multimodal expert behaviors via a structured latent variable while modeling long-range temporal dependencies within action chunks. All expert demonstrations are generated automatically using the RoboTwin 2.0 platform, which synthesizes trajectories under domain-randomized conditions. Unlike prior chunk-based methods such as ACT that rely on deterministic or unimodal generation, our contribution lies in a joint optimization framework that enables stable learning of latent-variable chunkwise policies under long-horizon visuomotor inputs. This framework consists of three core components: (1) the Spatio-Temporal Adaptive Augmentation (STAA) module, which dynamically adjusts augmentation strength across spatial and temporal dimensions; (2) the Adaptive Gradient Management Strategy (AGMS), which stabilizes optimization via percentile-based clipping and direction consistency checks; and (3) the Multi-Stage Learning Rate Scheduler (MLRS), which transitions the model through exploration, stabilization, and fine-tuning phases. Together with Progressive KL Regularization (PKL), these components enable the model to learn structured, temporally coherent action sequences directly from visual observations, improving both training stability and sequence-level consistency in long-horizon imitation learning.

We evaluate our method on RoboTwin 2.0, a large-scale bimanual manipulation benchmark designed for long-horizon visuomotor learning under domain-randomized settings. All evaluations are conducted using automatically generated expert demonstrations under identical training and inference conditions across methods. Policy performance is assessed through validation loss curves over 6,000 optimization steps and task success rates over 100 independent rollouts, with results averaged across multiple independent runs with different random seeds. Across all evaluated settings, our method consistently exhibits higher task success rates compared to baseline models, while incurring only minimal additional computational overhead.

## 2 Related work

### 2.1 Imitation learning and sequence modeling

Imitation learning (IL) has been widely adopted for acquiring manipulation skills from expert demonstrations. Early behavior cloning methods learn direct state-to-action mappings but suffer from error accumulation and distribution shift in long-horizon tasks (Florence et al., 2022; Mandlekar et al., 2021). Subsequent works address these issues through structured policies and sequence modeling that capture temporal dependencies in demonstrations (Gajewski et al., 2024; Janner et al., 2021; Paudel, 2022).

Recent advances reformulate robot control as sequence modeling using Transformers. Decision Transformer (Chen et al., 2021) and Trajectory Transformer (Kienle et al., 2024) demonstrate that autoregressive trajectory modeling enables effective long-horizon planning. Building on this, the Action Chunking Transformer (ACT) predicts multi-step action chunks rather than single-step actions, significantly reducing compounding errors in multi-task manipulation (Zhao et al., 2023). Similar chunk-based strategies have been explored in hierarchical IL and temporal abstraction frameworks (Koch et al., 2022; Wang et al., 2024; Le et al., 2018).

### 2.2 Multimodality and probabilistic policies

Despite their success, most Transformer-based IL methods rely on deterministic or unimodal action generation, limiting their ability to model the inherent multimodality of human demonstrations—where identical visual observations may correspond to multiple valid action sequences (Kim et al., 2019; Yao et al., 2025).

To address this, probabilistic generative models have been extensively employed. Conditional variational autoencoders (CVAEs) learn conditional distributions over trajectories and have been successfully applied to motion planning, grasp synthesis, and human–robot interaction (Ivanovic et al., 2020; Xu P. et al., 2025; Al-Kamil and Szabolcsi, 2024; Mérand et al., 2025). By introducing latent variables, these models capture multiple modes of expert behavior and improve robustness under ambiguous observations. Several works integrate CVAEs with recurrent or Transformer architectures for long-horizon action sequences (Dastider et al., 2025; Wang and Wan, 2019; Yao et al., 2025; Sermanet et al., 2024), with Transformer-based CVAE policies demonstrating improved diversity and expressiveness in complex tasks (Dimou et al., 2021; Xu B. et al., 2025). Related latent-variable formulations have also been explored for skill discovery and hierarchical control (Wu, 2025; Aita et al., 2025).

However, CVAE-based sequence models suffer from training instability issues-posterior collapse, degraded reconstruction, and hyperparameter sensitivity-especially under long-horizon prediction and long-horizon visual inputs. Recent advances in nonconvex and time-dependent optimization offer relevant insights. Coevolutionary neural dynamics navigate complex loss landscapes (Fan et al., 2025), while activated gradients improve convergence in deep networks (Liu et al., 2021). Distributed coordination frameworks stabilize joint optimization in multi-robot systems (Liu et al., 2024). For manipulator tasks, momentum recurrent dynamics enable efficient motion planning (Huang et al., 2025), and gradient-based differential solvers address time-varying constraints (Jin et al., 2022). Despite these advances, most CVAE-based imitation learning methods still rely on fixed KL weighting or manual annealing schedules, lacking adaptivity to training stage or task complexity. Thus, achieving stable optimization alongside expressive latent representations remains a central challenge.

### 2.3 Vision-language-action models and benchmarks

Concurrently, vision-based and vision-language-action (VLA) models have emerged as a dominant paradigm. End-to-end visuomotor policies trained from raw images show strong manipulation performance (Xie et al., 2025; Xu et al., 2022). Large Transformer-based VLA models such as PaLM-E (Driess et al., 2023), RT-1 (Brohan et al., 2022), RT-2 (Zitkovich et al., 2023), and OpenVLA (Kim et al., 2024) demonstrate impressive generalization across tasks and embodiments when trained on diverse multimodal datasets.

Parallel to model development, large-scale datasets and simulation platforms have been introduced to support data-driven IL. RoboNet (Dasari et al., 2019), BridgeData (Walka et al., 2023), Open X-Embodiment (Kembhavi et al., 2024), and RoboTwin 2.0 (Chen et al., 2025) provide multi-task demonstration datasets for systematic evaluation of long-horizon policies. RoboTwin 2.0, in particular, emphasizes twin-based data collection, temporal consistency, and multi-task coverage, making it a suitable benchmark for chunk-based and sequence-level IL methods. While these works demonstrate the potential of chunk-based and latent-variable policies, none systematically address the optimization instability specific to long-horizon visuomotor CVAE training. Our work fills this gap with a hierarchical training framework that jointly stabilizes latent learning and temporal prediction.

## 3 Method

In this work, we introduce the Spatio-Temporally Hierarchically Optimized Network (ST-HONet) for imitation learning, which enables robots to acquire robust and precise manipulation policies directly from long-horizon visual observations. All training data consist of automatically generated expert demonstrations from the RoboTwin 2.0 platform. Unlike prior chunk-based methods such as ACT, which combine CVAE with action chunking but suffer from training instability when learning long-horizon visuomotor policies, ST-HONet directly addresses three fundamental challenges: (i) fragile latent learning under visual domain shifts, (ii) gradient direction conflicts during long-sequence generation, and (iii) rigid KL annealing that fails to adapt to stage-wise training dynamics. To this end, we introduce a spatio-temporally hierarchical optimization framework that integrates three key mechanisms:

- **Spatio-Temporal Adaptive Augmentation (STAA):** A module that dynamically modulates the type and intensity of data augmentation across both spatial dimensions (appearance, occlusion, viewpoint) and temporal dimensions (frame dropping, interpolation, jittering) based on attention-derived weights.
- **Adaptive Gradient Management Strategy (AGMS):** A gradient stabilization mechanism that combines percentile-based clipping, direction consistency checks, and SNR-driven accumulation step adjustment to mitigate unstable optimization dynamics.
- **Multi-Stage Learning Rate Scheduler (MLRS):** A three-phase scheduler that transitions the model through exploration (cyclical LR), stabilization (cosine annealing), and fine-tuning (validation-loss-driven adaptation) stages based on the validation loss change rate.

**Figure 1:**
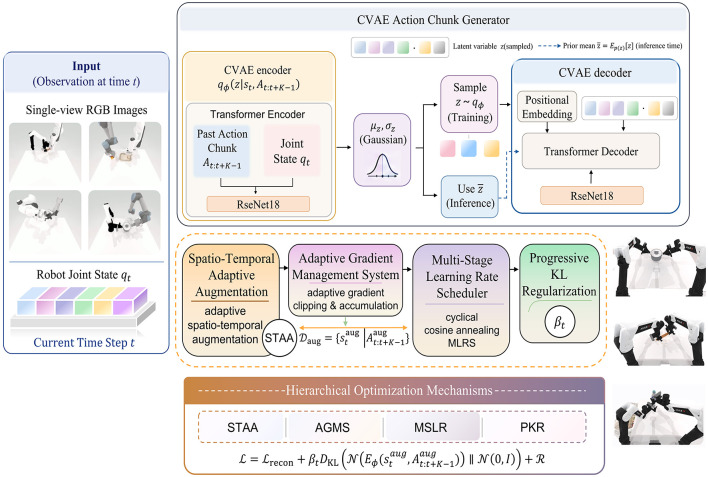
Architecture of the spatio-temporally hierarchically optimized network.

These components operate within a Transformer-based CVAE that generates action chunks conditioned on visual observations. The following subsections describe the CVAE formulation, the detailed design of each component, and their integration during training and inference (Figure 1).

During training, *z* is sampled from the posterior *q*_ϕ_(*z*∣*s, a*_*t*:*t*+*K*−1_), while at test time it is fixed to the prior mean $\overset{-}{z}=E\left[p\left(z\right)\right]$.

Within the imitation learning (IL) paradigm, a dataset of expert demonstration s = {(*s*_*t*_, *a*_*t*_)} is provided, and the objective is to learn a policy π_θ_(*a*_*t*_|*s*_*t*_) that reproduces expert behavior conditioned on the observed state. The Action Chunking Transformer extends this formulation by moving beyond single-step action prediction to directly modeling a sequence of future actions π_θ_(*a*_*t*:*t*+*k*_|*s*_*t*_), thereby explicitly modeling long- horizon temporal dependencies and reducing the error accumulation inherent in autoregressive, step-by-step rollout strategies.

We consider a setting in which a policy is learned from a corpus of human demonstration trajectories $D={\left\{\tau_{i}\right\}}_{i=1}^{N}$, the agent operates at time step *t* with an observation *s*_*t*_ = {*I*_*t*_, *q*_*t*_}, where *I*_*t*_ consists of multi-view RGB visual inputs and *q*_*t*_ denotes the robot's current joint posture. The objective is to infer a temporally extended action segment *a*_*t*:*t*+*K*−1_ = [*a*_*t*_, *a*_*t*+1_, …, *a*_*t*+*K*−1_], spanning *K* future time steps, with each action specifying target joint positions.

Human demonstrations inherently exhibit both long-range temporal structure and multimodality, as multiple valid action sequences may correspond to the same observed state. To capture these properties, we employ a Transformer-based conditional variational autoencoder (CVAE) as a hierarchical action sequence generator. The model parameterizes the conditional distribution *p*_θ_(*a*_*t*:*t*+*k*−1_|*s*_*t*_, *z*) , where the latent variable *z*~*p*(*z*) encodes high-level variations among distinct action patterns and enables stochastic yet structured sequence generation.

The encoder *E*_ϕ_ takes as input the current observation *s*_*t*_ together with the corresponding ground-truth action segment *a*_*t*:*t*+*K*−1_, and infers a latent representation by estimating the posterior distribution *q*_ϕ_(*z*|*s*_*t*_, *a*_*t*:*t*+*K*−1_). This posterior is parameterized as a Gaussian distribution with diagonal covariance, enabling efficient sampling and optimization in the latent space.

A Transformer-based decoder *G*_θ_ conditions on the observation *s*_*t*_ and the latent variable *z* to generate a predicted action segment â_*t*:*t*+*K*−1_. The latent variable is obtained either from the learned posterior or from the standard normal prior (z)=N(0,I), depending on the evaluation setting.

Model optimization is performed by maximizing the evidence lower bound (ELBO) Equation 1:


$$\begin{array}{l} L_{ELBO}=\\ E_{q_{\phi }\left(z|s_{t},a\right)}\left[\log p_{\theta }\left(a|s_{t},z\right)\right]-\beta •D_{KL}\left(q_{\phi }\left(z|s_{t},a\right)∥p\left(z\right)\right) \end{array}$$ (1)

Where the expectation term enforces accurate reconstruction of the action sequence, and the Kullback–Leibler divergence regularizes the latent distribution toward the prior.

We introduce the Spatio-Temporal Adaptive Augmentation mechanism that dynamically adjusts both the type and strength of data augmentation through a lightweight attention module, enabling adaptive control for data augmentation.

In this work, ‘spatial dimension' refers to within-frame visual augmentations (e.g., appearance perturbation, occlusion, viewpoint affine transforms), while ‘temporal dimension' refers to cross-frame augmentations applied to the action sequence (e.g., frame dropping, interpolation, temporal jittering). The hierarchy arises from the decoupled control of augmentation intensities via attention-derived spatial weight ρ_*t*_ and temporal weight τ_*t*_.

Given an observation sequence {*s*_*t*_, …, *s*_*t*+*K*−1_} and the corresponding action chunk *a*_*t*:*t*+*K*−1_, we first extract spatio-temporal features Equation 2:


$$\begin{array}{l} H_{t}=Encoder_{feat}\left(s_{t}\right),H\in {\mathbb{R}}^{K\times B} \end{array}$$ (2)

A lightweight spatio-temporal attention module computes an augmentation weight for each time step Equation 3:


$$\begin{array}{l} \rho_{t}=\sigma \left(W_{att}•AvgPool\left(H_{t}\right)+b_{att}\right) \end{array}$$ (3)

Where ρ_*t*_∈[0, 1] denotes the augmentation intensity for the *t*-th frame and σ(•) is the Sigmoid function.

Apply intensity-adjustable spatial enhancement to the input image:

Appearance perturbation Equation 4:


$$\begin{array}{l} I_{t}^{\prime }=I_{t}+\rho_{t}•\xi_{color},\xi_{color}N\left(0,\sigma_{c}^{2}\right) \end{array}$$ (4)

Structured occlusion Equation 5:


$$\begin{array}{l} M_{occ}Bernoulli\left(p_{occ}•\rho_{t}\right),I_{t}^{occ}=I_{t}⊙\left(1-M_{occ}\right) \end{array}$$ (5)

Viewpoint affine transformation Equation 6:


$$\begin{array}{l} T_{t}=\left[\begin{array}{c} r\left(\rho_{t}\right)\cos\phi \&-r\left(\rho_{t}\right)\sin\phi \&d_{x}\left(\rho_{t}\right)\\ r\left(\rho_{t}\right)\sin\phi \&r\left(\rho_{t}\right)\cos\phi \&d_{y}\left(\rho_{t}\right) \end{array}\right] \end{array}$$ (6)

Where the transformation magnitude *r*(•) and translations*d*_*x*_, *d*_*y*_ scale proportionally with ρ_*t*_.

Flip augmentation Equation 7:


$$\begin{array}{l} I_{t}^{flip}=Flip\left(I_{t};\rho_{t},axis\right) \end{array}$$ (7)

Where *axis*∈{*horizontal, vertical*}, and the flipping probability is defined as $p_{flip}=\rho_{t}\;{•}_{base}^{flip}$.

For the action sequence *A*_*t*:*t*+*K*−1_, we introduce a temporal attention weightτ_*t*_ extracted from *H*_*t*_ via a Temporal Convolutional Network Equation 8:


$$\begin{array}{l} \tau_{t}=Sigmoid\left(W_{time}•H_{t}+b_{time}\right) \end{array}$$ (8)

Where τ_*t*_∈[0, 1] represents the temporal augmentation intensity at time step *t*.

Random frame dropping and interpolation Equation 9:


$$\begin{array}{l} p_{drop}\left(t\right)=p_{0}•\tau_{t},\iota_{\nu }^{interp}=Lerp\left(\iota_{\nu_{s}},\iota_{\nu_{e}}\right) \end{array}$$ (9)

Temporal jittering Equation 10:


$$\begin{array}{l} \nu^{\prime }=\left(1+\kappa •\tau_{t}\right)•\nu +\varepsilon_{t},\kappa U\left(-0.1,0.1\right) \end{array}$$ (10)

Where the jitter magnitude is dynamically modulated by τ_*t*_.

To prevent excessive augmentation and ensure stable training, the STAA module is optimized using an auxiliary regularization loss Equation 11:


$$\begin{array}{l} L_{STAA}=\lambda_{r}∥\rho_{t}{∥}_{2}^{2}+\lambda_{t}∥\tau_{t}{∥}_{2}^{2}+\lambda_{e}H\left(\rho_{t},\tau_{t}\right) \end{array}$$ (11)

The augmented data are used to replace the original inputs for training the encoder *E*_ϕ_ and generator *G*_θ_ Equation 12:


$$\begin{array}{l} D_{aug}=\\ \left\{\left(s_{t}^{aug}=\left(I_{t}^{aug},q_{t}\right),A_{t:t+K-1}^{aug}=Aug_{time}\left(A_{t:t+K-1};\tau_{t}\right)\right)\right\} \end{array}$$ (12)

To address the gradient instability commonly observed in deep generative models, we adopt a percentile-based adaptive gradient clipping strategy, where the clipping threshold is dynamically adjusted based on recent gradient statistics Equation 13:


$$\begin{array}{l} c_{t}=percentile_{p}\left(\left\{∥g_{t-\tau }{∥}_{2},∥g_{t-\tau +1}{∥}_{2},\ldots ,∥g_{t-1}{∥}_{2}\right\}\right) \end{array}$$ (13)

Where *p* denotes the target percentile (typically set to 90–95), and τ is the size of the historical window.

The clipped gradient is computed as Equation 14:


$${\hat{g}}_{t}=\{\begin{array}{c} g_{t}\\ if∥g_{t}{∥}_{2}\leq c_{t}\\ \frac{c_{t}}{∥g_{t}{∥}_{2}}g_{t}\\ otherwise \end{array}$$ (14)

In addition to magnitude control, we introduce a gradient direction consistency check to detect unstable optimization dynamics Equation 15:


$$\begin{array}{l} sim_{t}=\frac{{ĝ}_{t}•{ĝ}_{t-1}}{∥{ĝ}_{t}{∥}_{2}∥{ĝ}_{t-1}{∥}_{2}},ifsim_{t}<\gamma \;for\;M\;consecutive\;steps,\;\\ \;then\;\eta_{t}=\eta_{t}•\delta \end{array}$$ (15)

Where η_*t*_ denotes the learning rate and δ is a decay factor. If *sim*_*t*_ < γ for*M* consecutive steps, the learning rate is decayed η_*t*_←η_*t*_•δ to maintain a larger step size when training is stable and to strengthen constraints when directions are chaotic.

For batches containing long temporal sequences, we compute gradient statistics using a sliding window Equation 16:


$$\begin{array}{l} \mu_{g}=\frac{1}{W}\overset{i}{\underset{j=i-W+1}{\sum }}g_{j},\sigma_{g}=\sqrt{\frac{1}{W}\overset{i}{\underset{j=i-W+1}{\sum }}{\left(g_{j}-\mu_{g}\right)}^{2}} \end{array}$$ (16)

Where *W* is the window size,

The gradient signal-to-noise ratio (SNR) is defined as *SNR*_*i*_ = ∥μ_*g*_∥_2_/σ_*g*_+,and then dynamically adjust the gradient accumulation steps accordingly Equation 17:


$$\begin{array}{l} N_{accum}^{\left(i\right)}=N_{base}•\left(1+\alpha •\tanh\left(\frac{SNR_{target}-SNR_{i}}{SNR_{scale}}\right)\right) \end{array}$$ (17)

Where *N*_*base*_ is the base accumulation step, α is a scaling coefficient, *SNR*_*target*_ is the desired SNR, and *SNR*_*scale*_controls sensitivity.

This allows the optimizer to increase accumulation under noisy gradients for smoother estimation, while reducing accumulation when gradients are reliable to accelerate updates.

The overall training process is divided into three stages:

- Stage I (Exploration): Model parameters are randomly initialized, requiring a relatively large learning rate to explore the parameter space.
- Stage II (Stabilization): The model begins to fit the data distribution and requires a smoothly decreasing learning rate.
- Stage III (Fine-tuning): The model approaches convergence and benefits from a small learning rate to refine action smoothness and precision.

Stage transitions are determined based on the validation loss change rate Equation 18:


$$\begin{array}{l} r_{t}=\frac{L_{val}\left(t\right)-L_{val}\left(t-\Delta \right)}{\Delta } \end{array}$$ (18)

where Δ denotes the evaluation window. Specifically,*r*_*t*_>ϵ_1_, exploration stage; ϵ_2_<*r*_*t*_ ≤ ϵ_1_−ϵ_2_,stabilization stage; *r*_*t*_ ≤ −ϵ_2_, fine-tuning stage.

Exploration stage: the Cyclical Learning Rate is used to enhance exploration Equation 19:


$$\begin{array}{l} \eta_{t}=\eta_{\min}+\frac{1}{2}\left(\eta_{\max}-\eta_{\min}\right)\left(1+\cos\left(\frac{T_{cur}}{T_{cycle}}\pi \right)\right) \end{array}$$ (19)

Where, η_min_ and η_max_ indicate the learning rate boundaries, *T*_*cur*_ denotes the current iteration step, and *T*_*cycle*_ is the cycle period.

Stabilization stage: the cosine annealing schedule is applied for smooth convergence Equation 20:


$$\begin{array}{l} \eta_{t}=\eta_{init}•\frac{1}{2}\left(1+\cos\left(\frac{T_{cur}}{T_{total}}\pi \right)\right) \end{array}$$ (20)

Fine-tuning stage: an adaptive learning rate driven by validation loss dynamics Equation 21:


$$\begin{array}{l} \eta_{t}=\eta_{t-1}•\exp\left(-\lambda •sign\left(r_{t}\right)•\min\left(|r_{t}|,r_{\max}\right)\right) \end{array}$$ (21)

Where λ controls sensitivity and *r*_max_ bounds the maximum adjustment.

In the CVAE framework, the KL divergence term regulates the alignment between the latent posterior and the prior. A fixed KL weight often fails to accommodate different training phases—over-constraining representation learning early on and risking posterior collapse in later stages.

We adopt progressive KL regularization, where the KL weight β is dynamically adjusted Equation 22:


$$\begin{array}{l} \beta_{t}=\beta_{\min}+\left(\beta_{\max}-\beta_{\min}\right)•\sigma \left(\frac{t-T_{transition}}{T_{scale}}\right) \end{array}$$ (22)

Where σ(•) denoting the Sigmoid function, *T*_*transition*_ the transition midpoint, and *T*_*scale*_ controlling the transition speed.

Additionally,we introduce KL divergence target scheduling, dynamically adjusting the target value based on the relative magnitude of the reconstruction loss and the KL divergence Equation 23:


$$\begin{array}{l} KL_{target}^{\left(t\right)}=KL_{base}•\left(1+\omega •\tanh\left(\frac{L_{recon}^{\left(t\right)}-L_{recon}^{\left(t-1\right)}}{L_{recon}^{\left(t-1\right)}}\right)\right) \end{array}$$ (23)

When reconstruction improvement slows, the KL constraint is relaxed to enhance expressiveness; when reconstruction improves rapidly, the KL constraint is strengthened to preserve latent structure.

The overall training objective integrates reconstruction loss, structured regularization, and auxiliary optimization terms Equation 24:


$$\begin{array}{l} L=L_{recon}+\beta_{t}D_{KL}\left(N\left(E_{\phi }\left(s_{t}^{aug},a_{t:t+K-1}^{aug}\right)\right)∥N\left(0,I\right)\right)+R \end{array}$$ (24)

The reconstruction loss combines multiple components Equation 25:


$$\begin{array}{l} L_{recon}=\omega_{smooth}L_{smooth-L1}\left(a,â\right)+\omega_{temp}L_{temporal-consistency}\\ \;\left(\Delta a,\Delta â\right)+\omega_{goal}L_{goal-aware}\left(a,â,s_{t}\right) \end{array}$$ (25)

The regularization term enforces motion smoothness and inter-block consistency Equation 26:


$$\begin{array}{l} R=R_{acc}+R_{boundary}=\omega_{acc}\overset{k-1}{\underset{i=2}{\sum }}∥a_{t+i+1}-2a_{t+i}+a_{t+i-1}{∥}_{2}^{2}\\ \;+\omega_{boundary}\overset{m}{\underset{j=1}{\sum }}∥a_{t+k}^{\left(j\right)}-a_{t}^{\left(j+1\right)}{∥}_{2}^{2} \end{array}$$ (26)

Where the acceleration regularization suppresses abrupt changes in motion dynamics, and the boundary consistency term ensures smooth transitions between consecutive action blocks, *K* denotes the action chunk length,*m* the number of overlapping chunks, and $a_{t+k}^{\left(j\right)}{,a}_{t}^{\left(j+1\right)}$ represent the terminal and initial actions of adjacent chunks, respectively.

Table 1 summarizes the overall training pipeline of the proposed method. The algorithm integrates adaptive data augmentation, latent-variable modeling, and multi-stage optimization to jointly improve training stability and long-horizon action prediction performance.

**Table 1:** ST-HONet training procedure.

Algorithm	Training
1: Given: Demo dataset *D*, chunk size *K*, Adaptive KL weight parameters	
2: Let: *s*_*t*_ = (*I*_*t*_, *q*_*t*_) represent the state at timestep *t*,*a*_*t*:*t*+*K*−1_ represent the action chunk, *z* represent the latent variable	
3: Initialize: Encoder *E*_ϕ_(*z*|*s*_*t*_, *a*_*t*:*t*+*K*−1_), Generator	
4: for iteration *n* = 1,2,…*n* = 1, 2,… do	
5: Sample *s*_*t*_and *a*_*t*:*t*+*K*−1_ from *D*	
6: Apply STAA to *s*_*t*_ and *a*_*t*:*t*+*K*−1_	
7: Encode: $\mu_{z},\sigma_{z}=E_{\phi }\left(s_{t}^{aug},a_{t:t+K-1}^{aug}\right)$	
8: Sample latent variable $zN\left(\mu_{z},\sigma_{z}\right)$	
9: Decode: ${Â}_{t:t+K-1}=G_{\theta }\left(s_{t}^{aug},z\right)$	
10: $L_{recon}$, R,$L_{KL}=D_{KL}\left(N\left(\mu_{z},\sigma_{z}\right)∥N\left(0,I\right)\right)$	
11: Update θ, ϕ with adaptive optimizer and $L=L_{recon}+\beta_{t}L_{KL}+R$	

## 4 Experiments

### 4.1 Experimental setup

**Experimental platform**. experiments are conducted on the RoboTwin 2.0 platform, which provides a unified simulation environment and a scalable benchmark for bimanual robotic manipulation. The robot is the Aloha-AgileX dual-arm system, comprising two 6-DOF manipulators each.

**Quantification of long-horizon tasks**. In this work, we operationalize “long-horizon” manipulation tasks with two quantitative descriptors. First, the episode length—the number of environment steps required to complete a task from initial state to termination—ranges from approximately 200 to 400 steps per episode, depending on task complexity. Second, the chunk-to-episode ratio, defined as the ratio between the action chunk size KK and the average episode length LL, is set to *K*/*L*≈1/4 to 1/8 (e.g., *K* = 50, *L*∈[200, 400]). This ratio implies that each action chunk covers a substantial portion of the full task horizon, requiring the policy to plan and coordinate over temporally extended subgoals rather than relying on frame-by-frame reactive control. These settings distinguish our benchmark from short-horizon tasks where chunk-to-episode ratios typically exceed 1/2.

**Data generation**. To obtain scalable yet behaviorally realistic training data, we adopt an automated data generation pipeline provided by RoboTwin 2.0. A small set of seed demonstrations (approximately 5 per task) is first collected via human teleoperation. Building on these seeds, the program-driven task execution pipeline of RoboTwin 2.0 automatically synthesizes an expanded dataset. Multimodal large language models (MLLMs) are prompted to generate executable task programs that mirror the structural patterns observed in the seed demonstrations. A simulation-in-the-loop refinement step, guided by vision-language model (VLM) feedback, ensures trajectory quality and diversity. This fully automated generation strategy produces 50 expert demonstrations per task under domain-randomized conditions. Critically, all models presented in this paper are trained exclusively on these automatically generated demonstrations, not on the raw human teleoperation data. Each demonstration includes multi-view RGB observations, robot joint states *q*_*t*_, and ground-truth action sequences.

#### 4.1.1 Task description

Beat Block Hammer: starting from initial poses, the robot must grasp a hammer, lift it, and strike a target block placed at a random location on the tabletop. Task success is defined by clear contact between the hammer head and the target block. The challenge arises from positional uncertainty induced by varying initial placements of both the hammer and the target block.

Open Laptop: starting from arbitrary lid angles, positions, and orientations, the robot must lift and fully open the laptop lid to a predefined target angle. The laptop's color is randomized across trials, visual cues are limited to the lid's edge and hinge region. The challenge lies in recovering from varied initial configurations while avoiding excessive force or slip.

**Figure 2:**
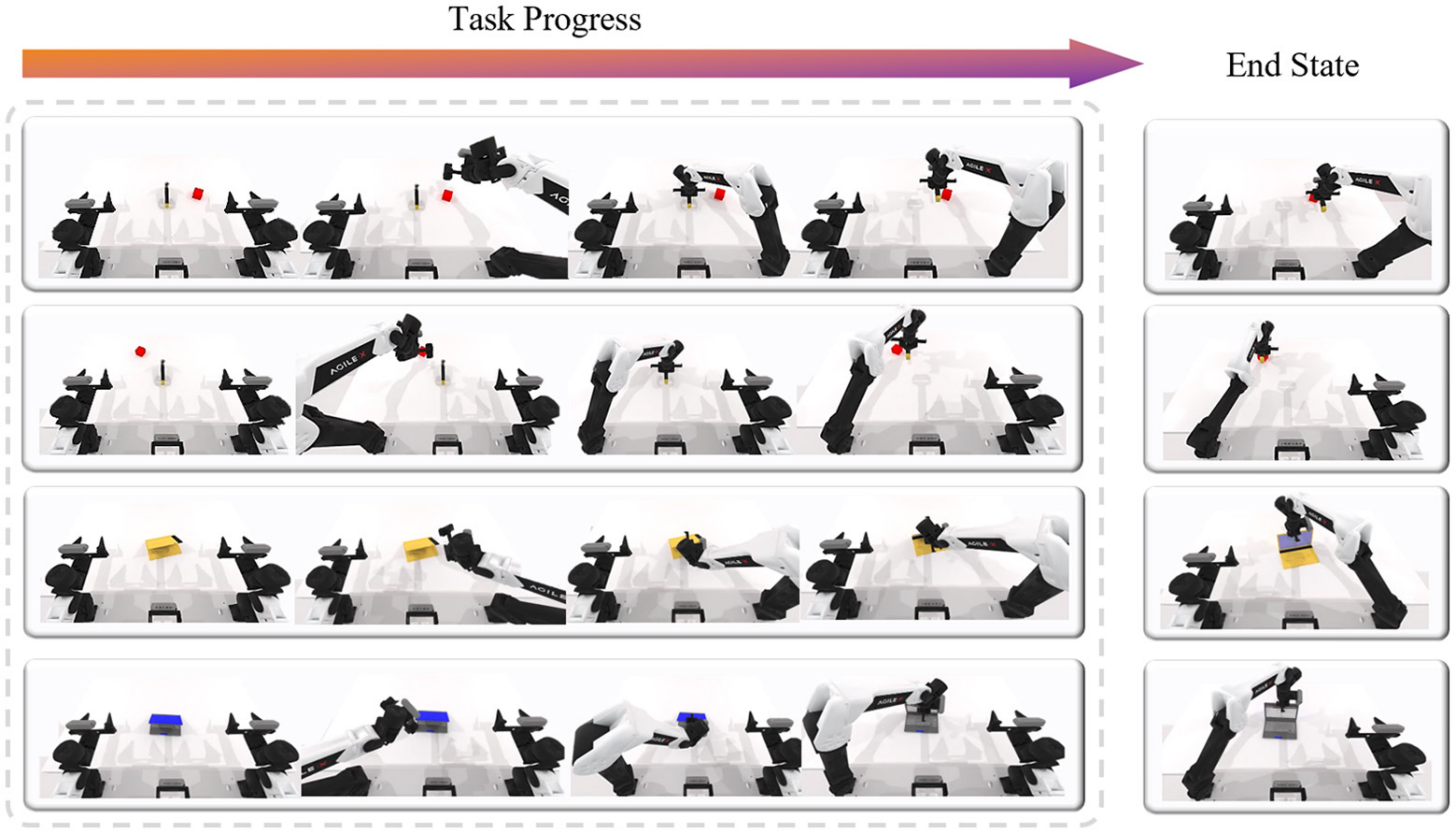
Task execution trajectories for Beat Block Hammer and Open Laptop.

The bimanual system autonomously selects the arm based on the target's relative workspace position (Figure 2).

#### 4.1.2 Evaluation protocol

To ensure a fair and reproducible evaluation, all methods are assessed under identical experimental conditions within the same simulated environment. For each task, we execute 100 independent rollouts and report the mean success rate. Success is determined by task-specific criteria defined in the benchmark, providing a rigorous and equitable assessment of policy robustness and generalization.

### 4.2 Model hyperparameters

To ensure reproducibility, this section details the hyperparameter configurations for the core model used in our experiments. The following table summarizes the key hyperparameter settings optimized for our dual-arm manipulation tasks (Table 2):

**Table 2:** Core hyperparameters of ST-HONet.

Category	Hyperparameter	Value
Model	Chunk size	50
	Hidden dimension	512
	Feedforward dimension	3,200
	Number of Transformer layers	6
	Number of attention heads	8
	State dimension	14
	Latent variable dimension	32
Training	Base learning rate	1 × 10^−5^
	Batch size	6
	Base KL weight	10.0
	Optimizer	AdamW
	Weight decay	1 × ^−4^
	Training steps	6,000
AGMS	Gradient clipping percentile	95%
	Direction consistency threshold	0.25
	Target SNR	1.0
MLRS	Exploration threshold	0.01
	Fine-tuning threshold	−0.001
	Cycle period	1,000 steps
PKL	KL weight range	0.1–20.0
	Transition midpoint	2,000 steps
STAA	Augmentation regularization weights	λ_*r*_ = 0.01, λ_*t*_ = 0.01, λ_*e*_ = 0.001
Loss	Reconstruction loss weights	ω_*smooth*_ = 1.0, ω_*temp*_ = 0.5, ω_*goal*_ = 0.3
	Regularization weights	ω_*acc* = 0.1_, ω_*boundary*_ = 0.2
Evaluation	Random seeds	5
	Rollouts per evaluation	100

Training Configuration Details:

- Learning Rate & Batch Size. A learning rate of 1 × 10^−5^ with a batch size of 6 was selected to ensure stable gradient updates while efficiently utilizing computational resources during training on the domain-randomized bimanual datasets.
- Chunk Size. The action prediction horizon is set to 50 time steps, enabling the model to plan sufficiently long sequences for complex, multi-stage manipulation tasks.
- Model Architecture. The transformer-based architecture employs a hidden dimension of 512 and a feedforward dimension of 3200. The state dimension (14) corresponds to the proprioceptive input from the dual-arm system, representing joint states for coordinated bimanual control.
- KL Weight. A KL divergence weight of 10 is applied in the training objective to balance reconstruction accuracy with latent space regularization.

### 4.3 Experimental results

#### 4.3.1 Validation loss analysis

**Figure 3:**
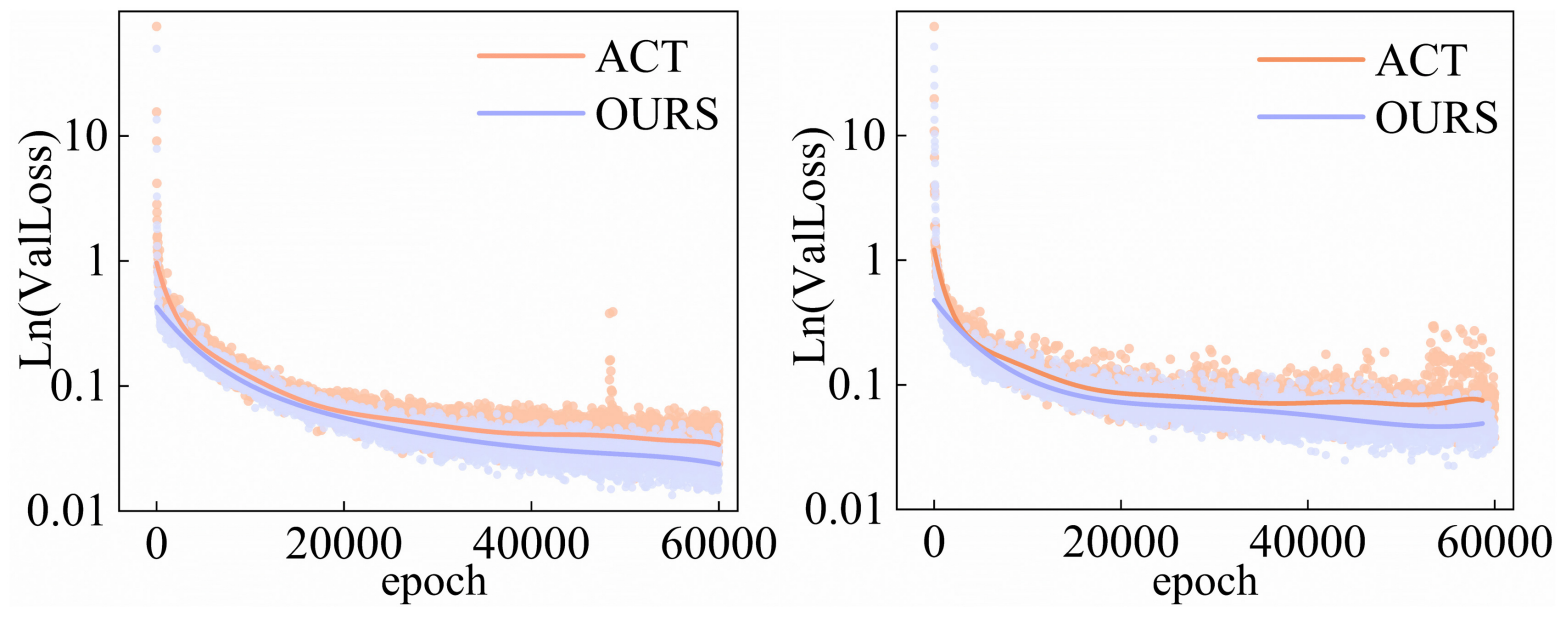
ValLoss of Beat Block Hammer and Open Laptop.

Figure 3 compares the validation loss curves of the ACT model and our method over 6,000 optimization steps for two representative tasks: Beat Block Hammer and Open Laptop. The y-axis is plotted on a logarithmic scale to clearly illustrate the loss decay dynamics. The figure includes validation losses from all stages alongside fitted curves derived from higher-order polynomials (Figure 3).

The proposed method exhibits consistently lower validation loss values and reduced step-to-step fluctuations compared to ACT. These results indicate improved optimization stability and smoother training dynamics. However, it should be noted that validation loss serves primarily as an indicator of training consistency rather than a direct measure of final policy quality. The primary evaluation of policy performance remains task success rate, reported in the following subsection.

**Computational complexity analysis**. We evaluate the computational efficiency of our method by comparing its memory usage and inference latency with ACT across two bimanual tasks (Table 3).

**Table 3:** Model complexity comparison.

Task	Model	Initial memory (GB)	Peak memory (GB)	Memory increment (GB)	Avg. inference time (s)
Beat Block Hammer	ACT	2.093	2.185	0.092	0.3987
	OURS	2.102	2.195	0.093	0.4558
Open Laptop	ACT	2.129	2.222	0.093	0.7437
	OURS	2.142	2.238	0.096	0.8998

In Beat Block Hammer, our approach shows only a marginal increase in both initial and peak memory, each under 0.5%, while memory increment remains virtually identical. For Open Laptop, memory overhead remains similarly low, with initial and peak memory differing by less than 0.8%, and memory increment increasing only slightly. Inference time exhibits a moderate but consistent increase: our method runs about 14% slower in Beat Block Hammer and 21% slower in Open Laptop.

Our approach introduces minimal memory overhead while delivering stronger task performance, maintaining a practical balance between robustness and real-time feasibility for bimanual manipulation.

**Figure 4:**
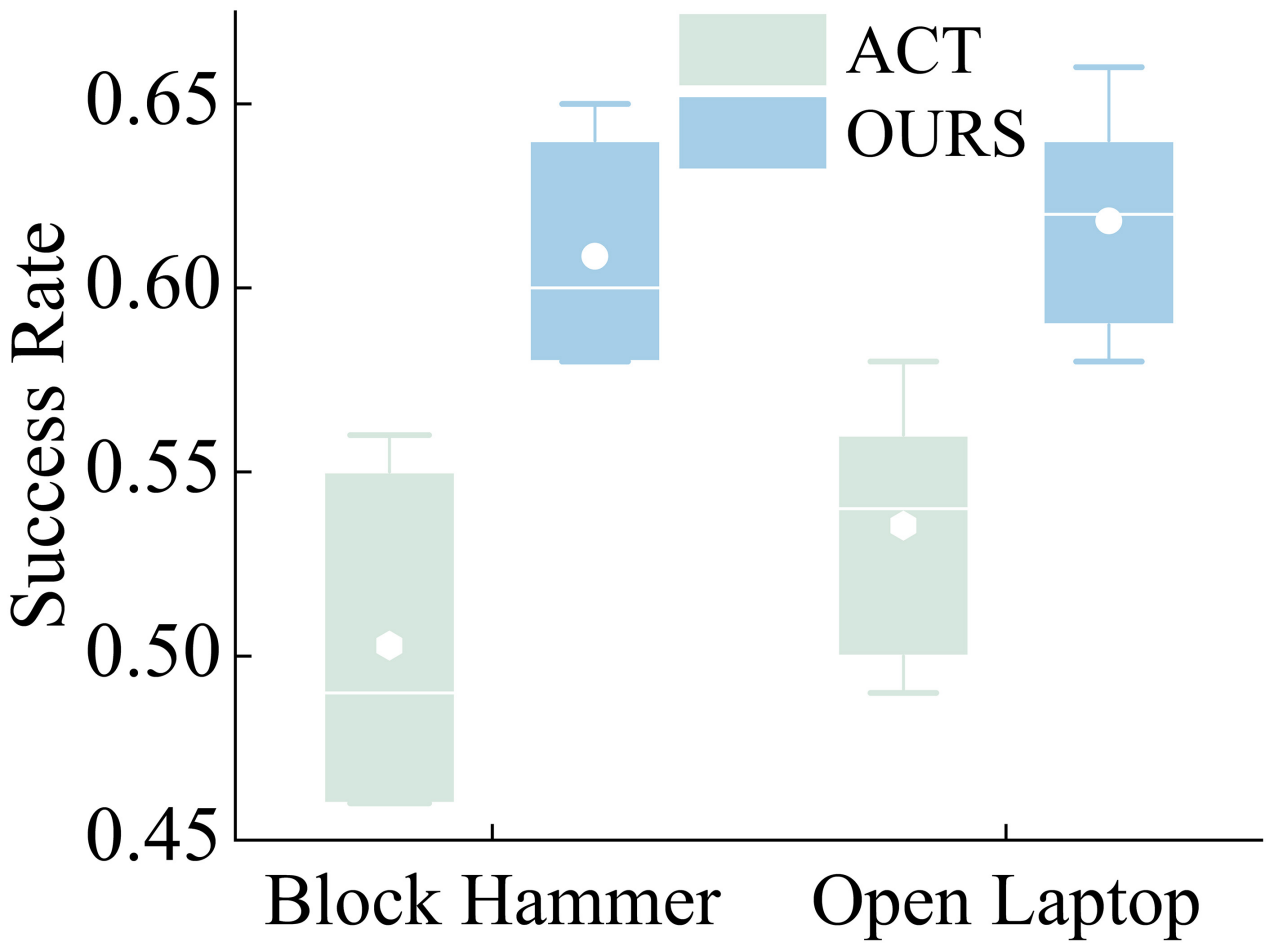
Inference success rate.

**Task success rate evaluation**. Figure 4 presents the main results of our evaluation: task success rates for models trained using the aforementioned method. Each task was evaluated using identical datasets and inference environments. We report success rates averaged over 100 independent rollouts per model, with five random seeds (Figure 4).

**Figure 5:**
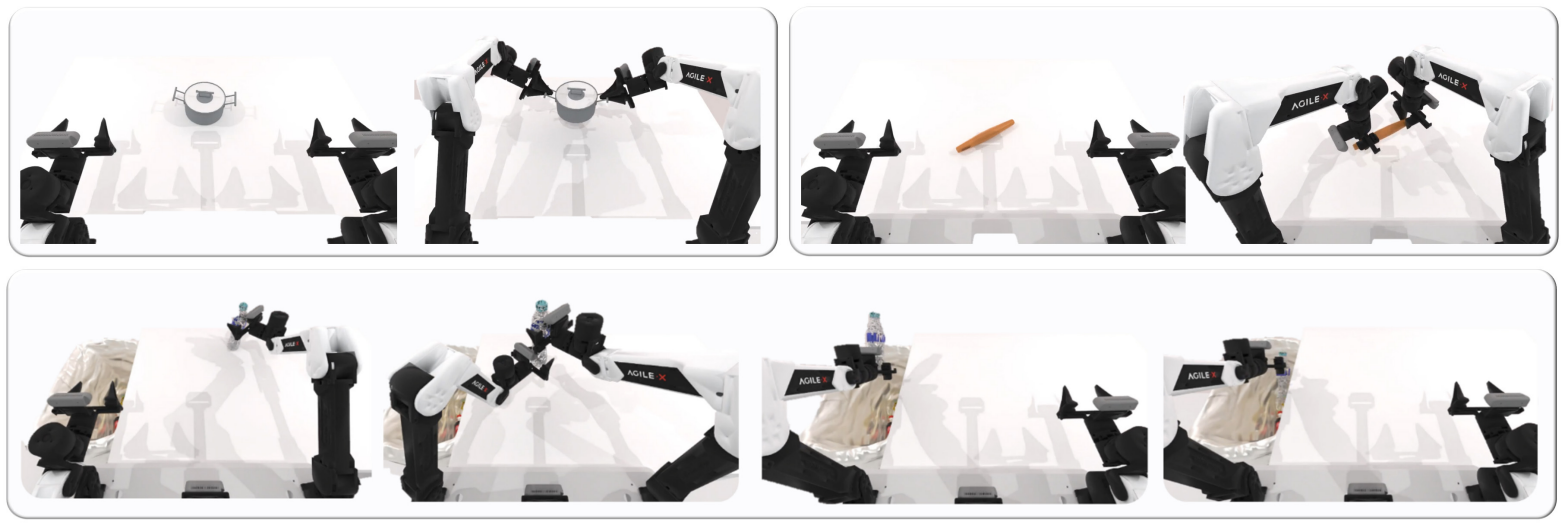
Illustration of representative manipulation tasks.

The proposed method achieves a maximum success rate of 0.65 on Beat Block Hammer, representing a 16.1% improvement over the baseline's 0.56. On Open Laptop, our method reaches 0.66, a 13.8% improvement over the baseline's 0.58. Our method consistently shows higher median success rates with lower variance, indicating enhanced stability and effectiveness in bimanual manipulation scenarios (Figure 5).

**Ablation studies**. To comprehensively evaluate the contribution of each proposed component under diverse manipulation demands, including both single-arm and bimanual coordination scenarios, we conduct ablation experiments on multiple representative tasks (Figure 5). We assess the individual contribution of each proposed component by incrementally adding STAA, AGMS, MLRS, and PKL to the baseline model across all tasks. All results are obtained from five independent training runs with different random seeds, and each trained model is evaluated over 100 rollouts (Table 4).

**Table 4:** Ablation study results (mean success rate ± std).

Configuration	Beat Block Hammer	Open Laptop	Lift Pot	Grab Roller	Put Bottles Dustbin
Baseline	0.50 ± 0.04	0.54 ± 0.05	0.80 ± 0.04	0.92 ± 0.04	0.29 ± 0.03
+ STAA	0.54 ± 0.05	0.58 ± 0.04	0.85 ± 0.04	0.94 ± 0.04	0.37 ± 0.04
+ AGMS	0.58 ± 0.03	0.60 ± 0.03	0.86 ± 0.03	0.95 ± 0.03	0.44 ± 0.05
+ MLRS	0.53 ± 0.06	0.57 ± 0.05	0.83 ± 0.05	0.93 ± 0.04	0.41 ± 0.04
+ PKL	0.56 ± 0.04	0.61 ± 0.04	0.89 ± 0.04	0.96 ± 0.03	0.49 ± 0.05
+ ALL (Full ST-HONet)	0.62 ± 0.03	0.64 ± 0.03	0.91 ± 0.03	0.98 ± 0.02	0.55 ± 0.03

The results demonstrate that each component contributes positively, with PKL and AGMS providing the largest individual gains. The full model achieves the highest success rate, confirming the synergistic effect of all four mechanisms (Table 5).

**Table 5:** Comparison with additional baselines (mean success rate ± std).

Model	Beat block hammer	Open Laptop	Lift Pot	Grab Roller	Put Bottles Dustbin
Pi0	0.43 ± 0.04	0.62 ± 0.04	0.84 ± 0.04	0.96 ± 0.04	0.52 ± 0.05
DP	0.42 ± 0.03	0.49 ± 0.05	0.39 ± 0.03	0.98 ± 0.04	0.24 ± 0.04
ACT	0.50 ± 0.04	0.56 ± 0.03	0.80 ± 0.04	0.92 ± 0.04	0.29 ± 0.03
ST-HONet (Ours)	0.62 ± 0.03	0.64 ± 0.03	0.91 ± 0.03	0.98 ± 0.02	0.55 ± 0.03

**Comparison with additional baselines**. We compare ST-HONet against two additional strong baselines: Diffusion Policy (DP) and Pi0. All models are trained and evaluated under identical conditions with five random seeds and 100 rollouts per seed. Across all tasks, ST-HONet achieves the highest success rates among all compared methods. We further conducted one-way ANOVA to compare the performance of all four methods on each task. The results revealed significant main effects of method on all tasks (*p* < 0.01 for each). Post-hoc Tukey HSD tests confirmed that ST-HONet significantly outperforms each baseline across all tasks (*p* < 0.05), with the largest effect sizes observed on bimanual coordination tasks (Cohen's d > 1.2).

## 5 Conclusion

In this work, we proposed ST-HONet, a spatio-temporally hierarchically optimized framework for long-horizon visuomotor imitation learning. By formulating policy learning as the conditional generation of temporally extended action segments, ST-HONet integrates a Transformer-based conditional variational autoencoder with a hierarchical optimization framework to explicitly capture the multimodality of human demonstrations. The framework centers on the joint design of adaptive augmentation, gradient-aware optimization, and progressive KL regularization, which together enable stable learning of multimodal chunkwise policies under long-horizon visuomotor inputs. To address the optimization challenges of long-horizon generative sequence modeling under long-horizon visual inputs, we introduced a unified spatio-temporal training framework that combines adaptive augmentation, progressive latent regularization, and multi-stage optimization. Experimental results on the RoboTwin 2.0 benchmark demonstrate that ST-HONet achieves consistently higher task success rates compared to baseline models, while incurring only minor additional computational overhead.

### 5.1 Limitations

While the proposed method shows promising results in simulation, several real-world challenges must be addressed for effective sim-to-real transfer. Perception noise, lighting variations, camera calibration errors, and unmodeled occlusions can degrade visual observations, violating the clean-input assumption of our spatio-temporal attention module. Actuation uncertainty (e.g., joint backlash, torque ripple, delayed response) may cause mismatches between predicted action chunks and actual robot motion, undermining temporal consistency. Contact dynamics (e.g., friction, deformation, impact) are often poorly captured by simulation, leading to unexpected force interactions absent in demonstration data. The simulation-to-reality mismatch in visual appearance, physical parameters, and latency remains a fundamental bottleneck. Future work will focus on domain adaptation techniques, including robust visual encoders pretrained on real-world data, online system identification for actuation parameters, and contact-aware policy fine-tuning with limited real-world rollouts.

## Data Availability

The raw data supporting the conclusions of this article will be made available by the authors, without undue reservation.
