# Correction: ST-HONet: Spatio-Temporal Hierarchical Network for long-horizon bimanual visuomotor imitation

**DOI:** 10.3389/fnbot.2026.1907235

**Published:** 2026-08-03

**Authors:** Xukun Liu, Fengjuan Xie, Kai Xu, Zhenyu Liu, Shenggang Wei, Guangning Li, Xu Sun

**Affiliations:** Northwest Institute of Mechanical and Electrical Engineering, Xianyang, Shaanxi, China

**Keywords:** bimanual manipulation, long-horizon visuomotor imitation learning, robot policy learning, spatio-temporally hierarchical optimization, variational action transformer

In the published article, there was an error in Table 1. Specifically, its text should be justified on both sides. A corrected Table 1 appears below.

**Table 1:** ST-HONet training procedure.

Algorithm	Training
1: Given: Demo dataset *D*, chunk size *K*, Adaptive KL weight parameters	
2: Let: *s*_*t*_ = (*I*_*t*_, *q*_*t*_) represent the state at timestep *t*,*a*_*t*:*t*+*K*−1_ represent the action chunk, *z* represent the latent variable	
3: Initialize: Encoder *E*_ϕ_(*z*|*s*_*t*_, *a*_*t*:*t*+*K*−1_), Generator	
4: for iteration *n* = 1,2,…*n* = 1, 2,… do	
5: Sample *s*_*t*_and *a*_*t*:*t*+*K*−1_ from *D*	
6: Apply STAA to *s*_*t*_ and *a*_*t*:*t*+*K*−1_	
7: Encode: $\mu_{z},\sigma_{z}=E_{\phi }\left(s_{t}^{aug},a_{t:t+K-1}^{aug}\right)$	
8: Sample latent variable $zN\left(\mu_{z},\sigma_{z}\right)$	
9: Decode: ${Â}_{t:t+K-1}=G_{\theta }\left(s_{t}^{aug},z\right)$	
10: $L_{recon}$, R,$L_{KL}=D_{KL}\left(N\left(\mu_{z},\sigma_{z}\right)∥N\left(0,I\right)\right)$	
11: Update θ, ϕ with adaptive optimizer and $L=L_{recon}+\beta_{t}L_{KL}+R$	

In the published article, a correction has been made to Section **4 Experiments**, subsection *4.1 Experimental setup*. In the first sentence of the first paragraph, the text “ll experiments” was erroneously misspelt. This has been corrected to “All experiments”, with the full sentence correctly reading: “All experiments are conducted on the RoboTwin 2.0 platform, which provides a unified simulation environment and a scalable benchmark for bimanual robotic manipulation.”

In the published article, in Section **4 Experiments**, the subheading “4.1.4 Policy training” was erroneously retained in the published article and should have instead been formatted as a bolded keyword at the beginning of the corresponding paragraph, as opposed to a third-level subsection heading. The heading levels of the following sections “4.1.5 Task description” and “4.1.6 Evaluation protocol” have been renumbered to “4.1.1 Task description” and “4.1.2 Evaluation protocol” accordingly and the corrected paragraph appears below.

**Policy training**. All models are trained exclusively on the automatically generated datasets, ensuring consistent and reproducible comparisons. For both tasks, we generate 50 expert demonstrations per task under domain-randomized conditions. The data format includes multi-view RGB observations, joint states, and action sequences.

In the published article, in section **4 Experiments**, the subheadings “4.3.1 Validation loss analysis,” “4.3.2 Computational complexity analysis,” “4.3.3 Task success rate evaluation,” “4.3.4 Ablation studies,” and “4.3.5 Comparison with additional baselines” were erroneously retained in the published article and should have instead been formatted as a bolded keyword at the beginning of the corresponding paragraph, as opposed to a third-level subsection heading. The corrected paragraphs appears below.

**Validation loss analysis. Figure 3** compares the validation loss curves of the ACT model and our method over 6,000 optimization steps for two representative tasks: Beat Block Hammer and Open Laptop. The y-axis is plotted on a logarithmic scale to clearly illustrate the loss decay dynamics. The figure includes validation losses from all stages alongside fitted curves derived from higher-order polynomials (Figure 3).

The proposed method exhibits consistently lower validation loss values and reduced step-to-step fluctuations compared to ACT. These results indicate improved optimization stability and smoother training dynamics. However, it should be noted that validation loss serves primarily as an indicator of training consistency rather than a direct measure of final policy quality. The primary evaluation of policy performance remains task success rate, reported in the following subsection.

**Computational complexity analysis**. We evaluate the computational efficiency of our method by comparing its memory usage and inference latency with ACT across two bimanual tasks (Table 3).

In Beat Block Hammer, our approach shows only a marginal increase in both initial and peak memory, each under 0.5%, while memory increment remains virtually identical. For Open Laptop, memory overhead remains similarly low, with initial and peak memory differing by less than 0.8%, and memory increment increasing only slightly. Inference time exhibits a moderate but consistent increase: our method runs about 14% slower in Beat Block Hammer and 21% slower in Open Laptop.

Our approach introduces minimal memory overhead while delivering stronger task performance, maintaining a practical balance between robustness and real-time feasibility for bimanual manipulation.

**Task success rate evaluation. Figure 4** presents the main results of our evaluation: task success rates for models trained using the aforementioned method. Each task was evaluated using identical datasets and inference environments. We report success rates averaged over 100 independent rollouts per model, with five random seeds (Figure 4).

The proposed method achieves a maximum success rate of 0.65 on Beat Block Hammer, representing a 16.1% improvement over the baseline's 0.56. On Open Laptop, our method reaches 0.66, a 13.8% improvement over the baseline's 0.58. Our method consistently shows higher median success rates with lower variance, indicating enhanced stability and effectiveness in bimanual manipulation scenarios (Figure 5).

**Ablation studies**. To comprehensively evaluate the contribution of each proposed component under diverse manipulation demands, including both single-arm and bimanual coordination scenarios, we conduct ablation experiments on multiple representative tasks (Figure 5). We assess the individual contribution of each proposed component by incrementally adding STAA, AGMS, MLRS, and PKL to the baseline model across all tasks. All results are obtained from five independent training runs with different random seeds, and each trained model is evaluated over 100 rollouts (Table 4).

The results demonstrate that each component contributes positively, with PKL and AGMS providing the largest individual gains. The full model achieves the highest success rate, confirming the synergistic effect of all four mechanisms (Table 5).

**Comparison with additional baselines**. We compare ST-HONet against two additional strong baselines: Diffusion Policy (DP) and Pi0. All models are trained and evaluated under identical conditions with five random seeds and 100 rollouts per seed. Across all tasks, ST-HONet achieves the highest success rates among all compared methods. We further conducted one-way ANOVA to compare the performance of all four methods on each task. The results revealed significant main effects of method on all tasks (*p* < 0.01 for each). *Post-hoc* Tukey HSD tests confirmed that ST-HONet significantly outperforms each baseline across all tasks (*p* < 0.05), with the largest effect sizes observed on bimanual coordination tasks (Cohen's d > 1.2).

The original version of this article has been updated.

